# The double-edged nature of antibody bivalency: mathematical and experimental analysis of cell surface antigen occupancy and opsonization

**DOI:** 10.1080/19420862.2026.2681843

**Published:** 2026-06-03

**Authors:** Luke Heirene, James Lodge, Marina Fedorova, Ross Gauntlett, Joanna Cordy, Zahra Rattray, Helen Byrne, Eamonn Gaffney, James W. T. Yates

**Affiliations:** a Mathematical Institute, University of Oxford, Oxford, United Kingdom; b Large Molecule Research, GSK, Stevenage, United Kingdom; c Strathclyde Institute of Pharmacy and Biomedical Sciences, University of Strathclyde, Glasgow, United Kingdom; d DMPK, Preclinical Sciences, GSK, Stevenage, United Kingdom

**Keywords:** Antibody, avidity, flow cytometry, mathematical model

## Abstract

Monoclonal antibodies are bivalent molecules and are thus able to engage two antigens concurrently, a property termed avidity. The therapeutic efficacy of an antibody drug can be broadly defined as a consequence of either antigen occupancy or target cell opsonization. Therefore, depending on the intended mechanism of action, avid engagement of a therapeutic antibody may be desirable in order to attain high antigen occupancy and consequent antagonism. In some cases, such as target cell opsonization and Fc-mediated cytotoxicity, avidity may limit efficacy when antigens are occupied with fewer antibodies per cell. In this study, we utilized a mathematical model of antibody-antigen binding to identify conditions under which avidity hinders or enhances therapeutic potential. We calibrated the model with *in vitro* assays exploring the binding of a bivalent and monovalent panel of anti-PD-1 antibodies with a range of affinities, against cell lines with a range of target densities. In calibrating the model to the *in vitro* binding data, we observed an affinity-dependent discrepancy between experimentally observed and model-predicted cell binding that we hypothesize arises due to unavoidable assay limitations. The calibrated model was then reused to correct for the assay bias and to generate refined estimates for on-cell antibody binding. The predictions generated by this model for the influence of avidity on cell surface receptor engagement with therapeutic antibodies may guide strategies for their structural engineering and dosing.

## Introduction

Central to the success of therapeutic antibodies is the ability to elicit biological effects through mechanisms such as direct antagonism, recruitment of immune effector cells, or internalization of antibody-drug conjugates (ADCs). Antagonism involves blocking signaling pathways by disrupting agonist (ligand) binding to cognate receptors, whereas immune effector function requires the activation of immune signaling cascades by stimulating Fc gamma receptor (Fc*γ*R)-mediated pathways, complement activation, or through other immunomodulatory receptors (e.g., CD3, NKp30).^1^ All of these mechanisms of antibody action require target engagement, with antibody immune effector function involving the formation of a ternary complex involving Fc*γ*Rs or complement. In particular, antagonist antibodies should be optimized to achieve high receptor occupancy, whereas antibodies that engage immune cells and ADCs may benefit from a greater density of bound antibody on the target cell surface.

The binding of an antibody to its cognate antigen, and consequent steric hindrance of receptor:ligand complex formation, can result in antagonism of associated signaling pathways. It follows that manipulating antigen occupancy is often a means to optimize therapeutic antibodies during early discovery. Antibody engagement of cell surface antigens is governed by the affinity of an antibody for the antigen, the valency by which it engages the antigen, and the density of the antigen on the cell surface.^2–4^ The affinity and valency of a therapeutic antibody can be modulated through a variety of engineering strategies, whereas the antigen density tends to remain fixed for a given cell line or disease indication.

For example, the affinity of a therapeutic antibody for its antigen is often manipulated through the use of directed evolution methods, introducing mutations at random in the complementarity-determining regions (CDRs) and measuring their impact on binding. Binding can be quantified using immobilized ligand-based or on-cell binding assays, but is most frequently reported as a 1:1 monovalent binding interaction using ligand-binding assays such as surface plasmon resonance (SPR).^5^

Beyond modulation of affinity, the valency of an antibody for its binding partners can also be engineered. Traditional monoclonal antibodies (mAbs) are bivalent for their antigens, but novel antibody engineering technologies enable heterodimerization and addition or reduction of binding or functional domains on the IgG scaffold, thus necessitating a greater understanding of how these covalently linked binding domains interact.^6^

Antigen density, by contrast, typically attains a defined magnitude for a given cell type, and in the context of therapeutic antibodies, different disease indications. Greater cell surface antigen density enables a greater potential level of antibody density on the cell surface, and associated decoration with immune-engaging moieties such as Fc domains and anti-CD3 domains. Consequently, greater antigen density has been associated with enhanced antibody effector function.^2^

**Figure 1.**
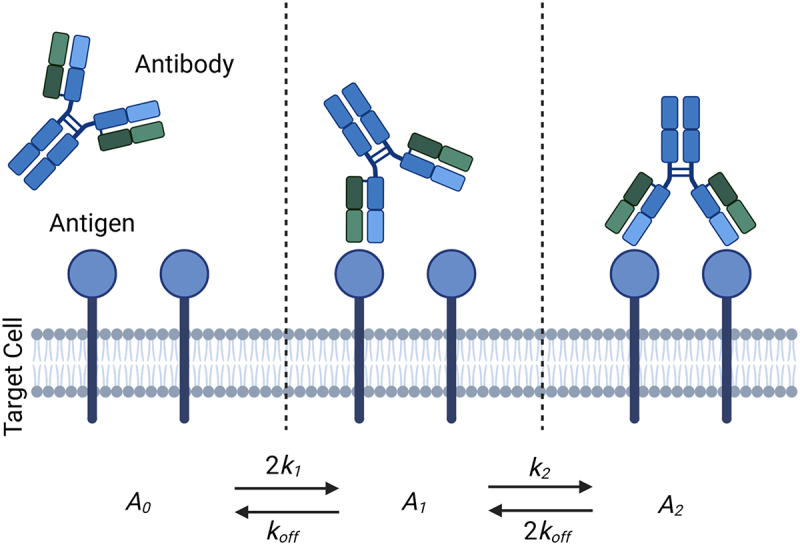
A schematic of antibody engagement with membrane-associated antigens. Initial association of unbound antibody (A_0_) with antigen occurs at a rate of k_1_ and forms the monovalent complex A_1_. The rate, k_2_, at which the bivalent complex A_2_ forms is enhanced by the spatial constraint introduced from the monovalent binding of the A_1_ species. Dissociation occurs at identical rates, k_off_, from A_2_ and A_1_ complexes. Rates are scaled based on their relative likelihood to occur; if two Fab domains are unbound, then the association rate for the antibody molecule is twice that of a single Fab binding. Figure created using Biorender.com.

The bivalent nature of mAbs enables them to engage up to two antigens concurrently, referred to as avidity.^7^ Bivalent engagement can increase the apparent affinity, as both Fab:antigen complexes must dissociate to release the antibody into solution (Figure 1). Experiments to measure antibody affinity for antigen by SPR are often designed to minimize avidity, despite the constrained nature of cell surface antigens, and the propensity for avidity to arise.^8^ Recent advanced methods have aimed to bridge this gap through novel chip preparation protocols,^9^ but the contribution of avidity to overall cell engagement otherwise remains poorly characterized. Advancements in the field have enabled the development of complex antibody-based formats that may contain additional binding domains, increasing their valency for one or more antigens, further necessitating an understanding of how multiple binding domains interact when covalently linked.

**Figure 2.**
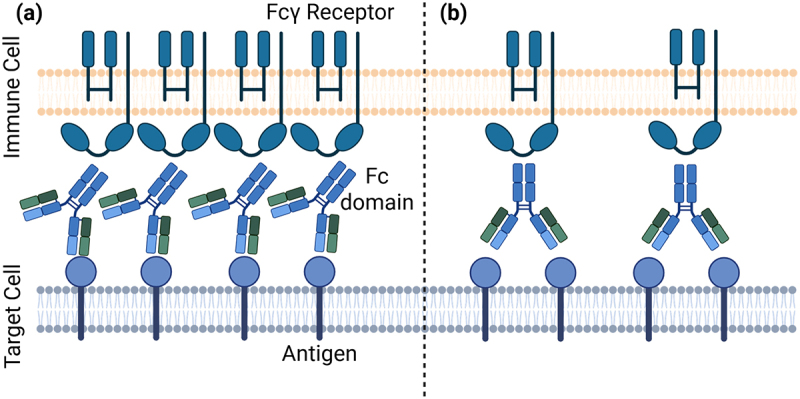
A schematic of antigen occupancy and Fc domain presentation following monovalent or bivalent antibody engagement. (a) Antibodies engaging antigens monovalently can achieve greater density and potentially achieve enhanced FcγR clustering. (b) In contract.

The enhanced stability of an avidly engaged antibody:antigen complex would be expected to result in enhanced antagonism compared to a monovalent interaction. Conversely, occupancy of two antigens with only a single Fc domain presented might limit Fc-mediated effector function through engagement with Fc*γ*Rs, reducing cytotoxic efficacy (Figure 2). This suggests that engineering a mAb to be monovalent may increase Fc*γ*R engagement at a given antibody concentration compared with the classical bivalent format. However, although a monovalent antibody has a greater potential to engage Fc*γ*Rs, the loss of avidity-mediated antigen binding may reduce the overall level of Fc*γ*R engagement. This effect is particularly pronounced when the number of antibodies or antigens is limited relative to Fc*γ*Rs, or when the antibody–antigen affinity is weak. Mathematical modeling can therefore be used to identify the conditions under which monovalent or bivalent antibodies achieve greater antigen binding or Fc*γ*R engagement.

There is increasing awareness of the role mathematical modeling can play in enhancing the development of antibody-based therapies, particularly in the context of avidity.^8,10–13^ Mathematical models, often formulated as ordinary differential equations (ODE), can be used to study how changes in antibody binding parameters, such as binding affinity or valency, affect therapeutic potential. Such ODE models, when combined with experimental data, can also be useful for testing and validating biological hypotheses.

The objective of this study was to use a mathematical model of bivalent antibody-antigen binding to examine the role of avidity in antibody-antigen binding and its potential effects on therapeutic outcomes. We explored the impact of the aforementioned antibody parameters, including affinity and valency for antigen, along with antigen density, in the model described in Heirene, et al.^11^, reporting both antigen occupancy and antibody density as outputs. We calibrated the model and its predictions against flow cytometry data relating to PD-1 binding profiles. This involved measurement of binding of a panel of antibodies targeting PD-1 with a range of affinities (*K*_*D*_ ≈1 − 100 nM), formatted as both bivalent and monovalent IgG-like molecules, to a panel of recombinant Chinese hamster ovary (CHO) cells expressing varying levels of PD-1. Three antibodies, in both monovalent and bivalent, for a panel of six antibodies were tested against four cell lines in total. In addition, we aimed to identify how mathematical modeling can enhance the interpretation of these assay measurements.

Furthermore, the relationship between SPR-derived dissociation constants (*K*_*D*_) and flow cytometry-derived half-maximal binding concentration (*EC*_50_) values is underexplored. Although these values are related, they each reflect the context of the assays from which they are derived. SPR experiments characterize 1:1 binding of an antibody to recombinant solubilized antigen on an artificial chip surface, and flow cytometry experiments characterize the same interaction with the antigen in its native membrane environment. In such a system using cells, avidity, internalization, and assay limitations such as ligand depletion and nonequilibrium binding can result in discrepancies between these two measurements of binding strength. Hence, we also aimed to more clearly establish the relationship between SPR *K*_*D*_ measurements and flow cytometry *EC*_50_ values.

In the sections below, we describe the mathematical model and experimental protocols and present the model simulation outputs, including how avidity might be expected to alter opsonization and antigen occupancy. We then calibrate this model using experimental data and discuss implications of these results for therapeutic antibody development and further work that could be undertaken to develop a greater understanding of antibody target engagement.

## Materials and methods

### Mathematical model

The mathematical model utilized in this study is an ODE model of bivalent, monospecific antibody-antigen binding within an *in vitro* binding assay. First introduced by Perelson and DeLisi^14^ and further developed by Heirene et al^11,12^, this model follows the reaction scheme in Figure 1. A free monospecific antibody ($A_{0}$) initially binds a target antigen with one arm to form a monovalent antibody–antigen complex ($A_{1}$). This complex may either dissociate, releasing the bound antigen, or bind a second free antigen with its unoccupied arm to generate a bivalent complex ($A_{2}$). Each antigen–antibody interaction is reversible, with both arms assumed to dissociate independently and at identical rates. We have assumed that there are no spatial gradients within the assay volume and that target antigens are distributed uniformly over the cell surface. We therefore choose ODEs to model the system, as the model variables are assumed to only evolve deterministically in time.

Applying the law of mass action to the reaction scheme shown in Figure 1, we have the following system of ODEs:

(1) $$\begin{array}{c} \frac{\mathrm{dr}}{\mathrm{dt}}=-2k_{1}rA_{0}+k_{\mathrm{off}}A_{1}-k_{2}rA_{1}+2k_{\mathrm{off}}A_{2} \end{array}$$

(2) $$\begin{array}{c} \frac{dA_{0}}{dt}=-2k_{1}rA_{0}+k_{\mathrm{off}}A_{1} \end{array}$$

(3) $$\begin{array}{c} \frac{dA_{1}}{dt}=2k_{1}rA_{0}-k_{\mathrm{off}}A_{1}-k_{2}rA_{1}+2k_{\mathrm{off}}A_{2} \end{array}$$

(4) $$\begin{array}{c} \frac{dA_{2}}{dt}=k_{2}rA_{1}-2k_{\mathrm{off}}A_{2} \end{array}$$

where the dependent variables are: the number of unbound target antigens, $r\left(t\right)$; the number of unbound antibodies, $A_{0}\left(t\right)$; the number of monovalently bound antibodies, $A_{1}\left(t\right)$; and the number of bivalently bound antibodies, $A_{2}\left(t\right)$. The factor of 2 that appears in certain reaction terms arises when two antibody arms can undertake a reaction (e.g., an antibody in solution can bind either of its arms and, similarly, when antibody is bivalently bound either arm may dissociate). To mimic the cell binding assay, Equations (1)-(4) are closed by imposing the following initial conditions:

(5) $$\begin{array}{c} r\left(0\right)=r_{\mathrm{tot}},A_{0}\left(0\right)=A_{\mathrm{tot}},A_{1}\left(0\right)=0,A_{2}\left(0\right)=0 \end{array}$$

In Equation (5), we assume that all antigens are initially unbound, and we denote by $A_{\mathrm{tot}}$ and $r_{\mathrm{tot}}$ the total number of antibodies and target antigens, respectively. The units of $A_{\mathrm{tot}}$ and $r_{\mathrm{tot}}$ are antibody number and cell surface receptor abundance, respectively. Due to the slow internalization rate of PD-1 and the relatively short duration of the assay, we neglect antigen internalization.^15^ Throughout the model, $r_{\mathrm{tot}}$ is viewed as a latent biological quantity, (e.g., receptors per cell or receptor density) in the governing equations. In theoretical simulations, we vary $r_{\mathrm{tot}}$ to explore how changes in receptor abundance affect predicted behaviors. When the model is subsequently linked to experimental measurements, the available receptor quantification readout is used as an empirical surrogate for $r_{\mathrm{tot}}$. Because the mapping from this readout to absolute receptor number is not independently established, experimentally assigned $r_{\mathrm{tot}}$ values should be interpreted in terms of relative differences (higher versus lower expression) and qualitative trends, rather than as quantitative receptor counts.

By taking suitable linear combinations of Equations (1)-(4) and leveraging Equation (5), it is straightforward to show that the total number of antibodies and antigens are conserved quantities within the system:

(6) $$\begin{array}{c} A_{0}+A_{1}+A_{2}=A_{\mathrm{tot}} \end{array}$$

(7) $$\begin{array}{c} r+A_{1}+2A_{2}=r_{\mathrm{tot}} \end{array}$$

We use Equations (6) and (7) to eliminate $A_{0}=A_{\mathrm{tot}}-A_{1}-A_{2}$ and $r=r_{\mathrm{tot}}-A_{1}-2A_{2}$ and, henceforth, we focus on the following reduced system for $A_{1}\left(t\right)$ and $A_{2}\left(t\right)$:

(8) $$\begin{array}{c} \frac{dA_{1}}{dt}=2k_{1}\left(r_{\mathrm{tot}}-A_{1}-2A_{2}\right)\left(A_{\mathrm{tot}}-A_{1}-A_{2}\right)-k_{\mathrm{off}}A_{1}\\ -k_{2}\left(r_{\mathrm{tot}}-A_{1}-2A_{2}\right)A_{1}+2k_{\mathrm{off}}A_{2} \end{array}$$

(9) $$\begin{array}{c} \frac{dA_{2}}{dt}=k_{2}\left(r_{\mathrm{tot}}-A_{1}-2A_{2}\right)A_{1}-2k_{\mathrm{off}}A_{2} \end{array}$$

with

(10) $$\begin{array}{c} A_{1}\left(0\right)=A_{2}\left(0\right)=0 \end{array}$$

In order to establish the impact of avidity, we compare simulations for bivalent and monovalent antibodies. As such, the analogous model for a monovalent antibody is

(11) $$\begin{array}{c} \frac{dA_{1}}{dt}=k_{1}\left(r_{\mathrm{tot}}-A_{1}\right)\left(A_{\mathrm{tot}}-A_{1}\right)-k_{\mathrm{off}}A_{1} \end{array}$$

with

(12) $$\begin{array}{c} A_{1}\left(0\right)=0. \end{array}$$

The monovalent model can be obtained by setting *k*_2_ = 0 and removing factors of 2 in Equations (8) and (9) while assuming $A_{2}\left(0\right)=0$.

Noting that assays within a reaction volume, $V_{\mathrm{well}}$, (litres, L), are used to estimate parameters, we estimate $A_{\mathrm{tot}}$, the number of antibodies within the system, for a given experimental antibody concentration from

(13) $$\begin{array}{c} A_{\mathrm{tot}}=A_{\mathrm{init}}\sigma \end{array}$$

Here, $A_{\mathrm{init}}$ is the initial antibody concentration (molar concentration, M), and *σ* is a “concentration-to-antibody-number” conversion factor given by

(14) $$\begin{array}{c} \sigma =\frac{V_{\mathrm{well}}\mathrm{Na}}{T^{0}} \end{array}$$

where *Na* = 6.02214 × 10^23^ is Avogadro’s constant (mol^−1^) and $T^{0}$ is the number of target cells within the reaction volume. Equation (14) is normalized with respect to $T^{0}$ to consider binding for a single target cell.

The in-solution binding rate, *k*_on_, is typically reported in the literature with units of (Ms)^−1^. In this work, however, we model binding in terms of absolute numbers of antibodies and antigens rather than concentrations. Accordingly, we rescale *k*_on_ so that its units are consistent with those used for $A_{1}$ and $A_{2}$:

(15) $$\begin{array}{c} k_{1}=\frac{k_{\mathrm{on}}}{\sigma } \end{array}$$

where the units of *k*_1_ are the number of antibody per second. All model simulations were performed in Python using Scipy’s *solve_ivp* function^16^ and the parameter values stated in Supplementary Table S1.

### Expressions for impact of avidity on EC_50_ and EC_90_

A particular focus of this work is to investigate the impact of avidity on the pharmacological parameters, *EC*_50_ and *EC*_90_ under equilibrium binding conditions. These quantities are defined as the antibody concentrations required to achieve 50% and 90% of maximum binding, respectively, where binding is measured in terms of either antigen occupancy or antibody opsonization. Using the mathematical models for bivalent and monovalent antibody binding Equations (8), (9) and (11), we derive analytic expressions for *EC*_50_ and *EC*_90_. These are presented below (see Supplementary information Section S1 for details).

For a bivalent antibody, we define $EC_{\phi }^{bo\mathrm{und}}$, the concentration at which the fraction *ϕ* of maximum surface-bound antibody is achieved, as follows

(16) $$\begin{array}{c} EC_{\phi }^{bo\mathrm{und}}=\frac{K_{D}}{2}\left(\frac{\phi r_{\mathrm{tot}}-f_{1}\left(\phi \right)}{r_{\mathrm{tot}}\left(1-\phi \right)-f_{1}\left(\phi \right)}\right)+\frac{\phi r_{\mathrm{tot}}}{\sigma } \end{array}$$

where *K*_*D*_ = *k*_off_*/k*_on_ = *k*_off_*/*(*σk*_1_) is the dissociation constant, and

(17) $$\begin{array}{c} f_{1}\left(\phi \right)=\frac{r_{\mathrm{tot}}+\frac{2k_{\mathrm{off}}}{k_{2}}-\sqrt{{\left(r_{\mathrm{tot}}+\frac{2k_{\mathrm{off}}}{k_{2}}\right)}^{2}-4\phi r_{tot}^{2}\left(1-\phi \right)}}{2} \end{array}$$

For example, *EC*_50_^bound^ and *EC*_90_^bound^ are determined by setting *ϕ* =0.5 and *ϕ* =0.9, respectively, in Equation (16).

When binding is instead defined in terms of antigen occupancy, we use the following expression, $EC_{\phi }^{occ}$, where

(18) $$\begin{array}{c} EC_{\phi }^{occ}=\frac{K_{D}}{2}\left(\frac{\phi r_{\mathrm{tot}}-2f_{2}\left(\phi \right)}{r_{\mathrm{tot}}\left(1-\phi \right)}\right)+\frac{\phi r_{\mathrm{tot}}-f_{2}\left(\phi \right)}{\sigma } \end{array}$$

with

(19) $$\begin{array}{c} f_{2}\left(\phi \right)=\frac{\phi r_{tot}^{2}\left(1-\phi \right)}{\frac{2k_{\mathrm{off}}}{k_{2}}+2r_{\mathrm{tot}}\left(1-\phi \right)} \end{array}$$

For a monovalent antibody, $f_{1}\left(\phi \right)=0$ and Equation (16) is replaced by

(20) $$\begin{array}{c} EC_{\phi }=\phi \left(\frac{K_{D}}{1-\phi }+\frac{r_{\mathrm{tot}}}{\sigma }\right) \end{array}$$

To quantify the impact of avidity on these pharmacological measures, we define

(21) $$\begin{array}{c} \mathrm{\Delta E}C_{\phi }^{bo\mathrm{und}}=\log_{10}\left(\frac{EC_{\phi }}{EC_{\phi }^{b\mathrm{ound}}}\right) \end{array}$$

(22) $$\begin{array}{c} \mathrm{\Delta E}C_{\phi }^{occ}=\log_{10}\left(\frac{EC_{\phi }}{EC_{\phi }^{occ}}\right) \end{array}$$

These quantities capture the 10-fold change in antibody concentration needed for monovalent and bivalent antibodies to achieve the same level of bound antibody and similarly for antigen occupancy.

### Stable cell line generation

Four recombinant CHO cell lines expressing varying densities of PD-1 were generated by co-transfecting ATUM Leap In® transposase mRNA, with ATUM vectors containing PD-1 under one of four promoters (EF1*α*, PGK, Tet responsive promoter or SV40D3). Host CHO GSKO suspension cells were maintained in CD CHO medium (Thermo Scientific 10,743,029) supplemented with 8 mM L-Glutamine (Thermo Scientific 25,030,024). Cells were transfected using FreeStyle™ MAX transfection reagent (Thermo Scientific 16,447,100) according to the manufacturer’s instructions. Briefly, 9 × 10^6^ cells (1 × 10^6^ cells/mL) were co-transfected with 2.5–9 *µ*g plasmid DNA and 2.7 *µ*g Leap In® transposase mRNA in the presence of 15 *µ*L FreeStyle™MAX reagent. Following transfection, cultures were incubated under standard shaking conditions (125 RPM, 37°C, 5% CO_2_, and 80% humidity). Selection was initiated 24–48 hours post transfection using either glutamine synthetase (GS) selection for GS-based vectors (CD CHO media supplemented with 50 *µ*M L-Methionine sulfoximine (MSX, Sigma GSS-1015-F), or Geneticin™ for neomycin resistance vectors (CD CHO media supplemented with 8 mM L-Glutamine and 500 *µ*g/ml G418 Sulfate (Thermo Scientific 10,131,027). Cell viabilities and densities were examined every 3–4 days before passaging the cells into fresh selection media. Stable cellular pools were characterized by flow cytometry. Due to heterogeneity observed in the Tet promoter pool, this cell line was subsequently subjected to single cell cloning using the CellCelector™ Flex instrument (Sartorius) to isolate a uniform clonal line.

### Antigen quantification

The cell surface density of PD-1 was determined by flow cytometry, comparing fluorescence of immunoprobed recombinant CHO cells against a standard curve generated using beads with known antibody binding capacity stained with the same fluorescent anti-PD-1 antibody. Recombinant CHO cells, stably expressing PD-1, were incubated for 30 minutes in the presence of allophycocyanin (APC)-conjugated mouse anti-human PD-1 antibody (BioLegend)(1:10) supplemented with 0.5% w/v BSA (Sigma-Aldrich). In parallel, a Quantum Simply Cellular anti-mouse kit (Bangs Laboratories) with pre-defined numbers of antibody binding sites was incubated with the same antibody staining solution as per manufacturer’s instructions.

All samples were washed twice prior to resuspension, and the median fluorescence intensity (MFI) was recorded in the APC channel (660/20) on a Cytoflex S (Beckman Coulter). MFI of Quantum Simply Cellular beads was plotted against manufacturer-supplied binding site numbers to generate a linear regression. The MFI from stained cells was interpolated against the linear regression to calculate the number of binding sites, assuming a 1:1 stoichiometry of antibodies to sites.

### Generation of antibodies

DNA fragments encoding variable heavy (VH) and variable light (VL) sequences for proprietary anti-PD-1 antibodies were cloned into IgG1 × 01 heavy and light chain sequences in the pTT5 vector, which also contained heterodimerization mutations that drive the desired heavy-chain pairing. DNA was pre-incubated with PEIpro (Sartorius 101,000,026) transfection agent for 10 minutes and transfected into HEK293-6E cells (NRC Canada) in BalanCD HEK293 growth media (Irvine Scientific 91,165) at a density of 2 × 10^6^ cells/mL.

BalanCD HEK293 media was modified to include 0.05% Geneticin™Selective Antibiotic (v/v) (50 mg/mL, ThermoFisher Scientific 10,131–027) and 2% GlutaMAX-1 (v/v) (100X, ThermoFisher Scientific 35,050,061). Cell culture conditions for antibody expression were performed in a TubeSpin BioReactor 600 (TPP 87,600) under agitation 230 RPM, 37°C, 5% CO_2_ (v/v) and 70% relative humidity in a Multitron II shaking incubator (Infors HT).

**Figure 3.**
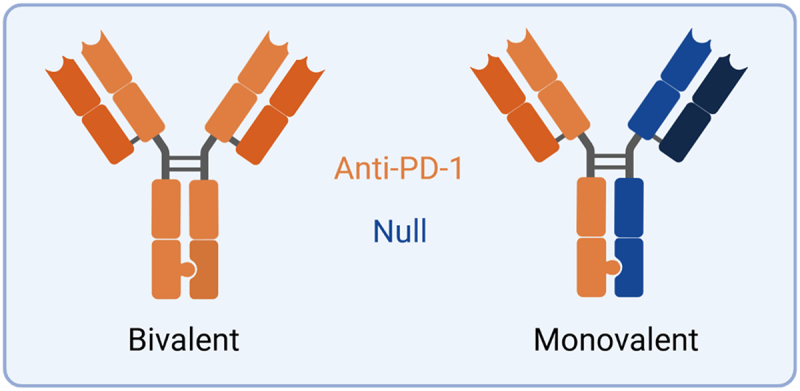
A schematic of the antibody formats generated for this work. Bivalent antibodies possess two identical anti-PD-1 VH and VL domains (orange). Only one half of the monovalent antibodies possess these VH and VL domains; the other side contains those of a non-PD-1 targeting antibody (blue). Figure created using Biorender.com.

Cell® culture feeding was performed on day three post-transfection with 10 mL of Tryptone N-1 (200 g/L in BalanCD HEK293 growth media, Organotechnie 19,533), and 10 mL of Fructose (540 g/L in BalanCD HEK293 growth media, Sigma Aldrich F0127) on day four post-transfection. Seven days post-transfection, antibody material was purified from transfected cell culture supernatant using Mag Sepharose™PrismA bead resin (Cytiva 17,550,001), prior to buffer exchange into phosphate-buffered saline (PBS). Half-antibody chains were mixed in equal volumes of equimolar solutions, and exposed to 25 mM cysteamine (Sigma Aldrich M9768) for 2 hours at 30°C. Bivalent antibodies possessed identical VH and VL domains on both mixed species, whereas monovalent constructs possessed VH and VL sequences from a non-PD-1-targeting antibody (Figure 3). Buffer exchange was performed once more in PBS, prior to confirmation of species identity by mass spectrometry.

### Determination of antibody binding kinetics

SPR experiments were performed using a Biacore 8K+ (Cytiva) at 25°C with HBS-EP+, pH 7.4 (Teknova H8022) as the running buffer. Recombinant Protein A (50 *µ*g/mL, Sigma-Aldrich P3838) diluted in 10 mM Sodium Acetate pH 4.5 (Cytiva BR100350) was amine-coupled to a CM5 sensorchip (Cytiva 29,149,603), according to the manufacturer’s protocol and amine coupling kit (Cytiva, BR100050). Test antibodies (2.04–14.14 *µ*g/mL, in HBS-EP+, pH 7.4) were captured onto the Protein A sensorchip (10 *µ*L/min, 60 seconds) and soluble PD-1 (in-house, diluted in HBS-EP+, pH 7.4) was flowed over the surface at increasing concentrations from 0–100 nM in a four-fold serial dilution series (300s association, 600s dissociation at 30 *µ*L/min flow rate). Buffer only (HBS-EP+, pH 7.4) was used as a blank control to measure any nonspecific binding of the soluble PD-1 to the Protein A sensorchip and a non-PD-1-targeting antibody was a negative control to measure any nonspecific binding of PD-1 to the antibody framework. The sensorchip surface was regenerated using two 15s injections of 10 mM glycine, pH 1.5 (in-house, 30 *µ*L/min) following each cycle. Sensorgrams were fitted to a 1:1 binding model with *R*_*max*_ local, from which kinetic rate constants (*k*_on_ and *k*_off_) and affinity (*K*_*D*_) was determined using the Biacore Insight Evaluation software v5 (Cytiva).

### Cell binding assays

Titrations of antibodies (2.09 × 10^−13^ − 1 × 10^−6^ M), prepared in an assay buffer (PBS (Gibco 14,190,144) supplemented with 0.5% v/v bovine serum albumin (Sigma Aldrich A7979) and 2 mM EDTA (Sigma Aldrich E7889), were incubated with recombinant CHO cell lines possessing varying densities of human PD-1 for one hour at 25°C. Supernatant was then aspirated, and cells were washed with assay buffer twice, and APC-conjugated goat antihuman IgG (1:50 in assay buffer, Jackson ImmunoResearch 109–136-127) was added to all samples for one hour at 25°C. The supernatant was aspirated, and cells were washed again in assay buffer. Median APC fluorescence intensity was measured using an iQue® 3 VBR flow cytometer (Sartorius, Göttingen, Germany).

### Model parameter estimation from experimental data

We now use our mathematical model of antibody-antigen binding to estimate unknown parameters through Bayesian inference. We generated *in vitro* cell binding data for three antibody clones with Fab arms of differing affinity in both bivalent and monovalent format (totaling six antibodies). In addition, antibody binding was generated for recombinant CHO cell lines expressing varying densities of PD-1. To mitigate poor signal-to-noise ratio for weaker affinity antibodies, binding data for all antibodies was fitted simultaneously for each cell line. For each antibody clone of differing affinity, j, ($j\in \left\{1,2,3\right\}$), the number of bound antibodies from the experimental data is fitted to the mathematical model. We fitted the model for both bivalent and monovalent formats using the following equations:

(23) $$\begin{array}{c} Y_{j}^{biv}=C_{j}^{biv}X_{j}^{biv},Y_{j}^{\mathrm{mono}}=C_{j}^{\mathrm{mono}}X_{j}^{\mathrm{mono}} \end{array}$$

where $Y_{j}^{biv}$ and $Y_{j}^{\mathrm{mono}}$ are 3 × *n* matrices of experimental binding values (*n* is the number of antibody concentration data points), $X_{j}^{biv}$ and $X_{j}^{\mathrm{mono}}$ are the corresponding 3 × *n* matrices of model predictions, and $C_{j}^{biv}$ and $C_{j}^{\mathrm{mono}}$ the fitted proportionality constants that relate experimental to simulated bound antibody number for a given affinity clone in bivalent and monovalent format, respectively. The complete system can be expressed in block-matrix form as

(24) $$\begin{array}{c} Y=\underset{\left(6\times n\right)}{\left[\begin{array}{c} Y^{\mathrm{biv}}\\ Y^{\mathrm{mono}} \end{array}\right]}=\underset{\left(6\times 6\right)}{\left[\begin{array}{c} C^{\mathrm{biv}}\\ C^{\mathrm{mono}} \end{array}\right]}\underset{\left(6\times n\right)}{\left[\begin{array}{c} X^{\mathrm{biv}}\\ X^{\mathrm{mono}} \end{array}\right]} \end{array}$$

The last three columns of the matrix $C^{\mathrm{biv}}$ are padded with zeros while the non-zero entries within a given row are the same. Similarly, the first three columns of $C^{\mathrm{mono}}$ are padded with zeros with the non-zero entries for a given row also being the same. Ultimately, we aim to identify the bivalent binding rate *k*_2_ and the vectors $C^{\mathrm{biv}}$ and $C^{\mathrm{mono}}$ for each cell line with all other parameters fixed.

Parameter inference is performed using Markov chain Monte Carlo (MCMC) with Haario-Bardenet adaptive covariance and three chains using the Python package PINTS.^17^ We assume the data follow a Gaussian error distribution, consistent with common practice in quantitative systems modeling where measurement noise arises from multiple independent sources. In flow cytometry, variability reflects a combination of photon-counting noise, fluctuations in detector sensitivity, cell-to-cell biological heterogeneity, and sample handling variation; because these contributions act additively on the measured fluorescence intensity, the central limit theorem supports approximating their combined effect with a normal distribution. This Gaussian likelihood is therefore an appropriate and tractable choice for Bayesian inference, particularly when residuals appear symmetric and approximately homoscedastic across the measured intensity range. We also estimate the standard deviation of the Gaussian error distribution during inference. We set the maximum number of MCMC iterations to 2 × 10^4^ and remove the first 5000 iterations when producing the final posterior parameter distributions to account for the burn in period where the chains have not yet converged. During inference, we aim to minimize the mean-squared error between model output and experimental data.

## Results

To highlight the influence of valency on the predicted number of bound antibodies and antigen occupancy, model simulations were performed to compare monovalent and bivalent antibody binding. We then calibrated the model using *in vitro* antibody binding data across different binding affinities and PD-1 densities. In previous work, we predicted that the second binding rate, $k_{2}$, is limited by antigen surface diffusion.^11^ Since $k_{2}$ depends on the antigen surface diffusion coefficient, we adopt a representative value of $D=10^{-14}\;m^{2}s^{-1}$, which yields $k_{2}=10^{-4}\;s^{-1}$; this value is used in the numerical simulations in the results section. However, other factors such as steric constraints may alter the value of $k_{2}$ in a given experimental system, so we estimate its value when fitting from the data in a later section.

### Avidity creates a barrier for the number of cell surface bound antibodies

**Figure 4.**
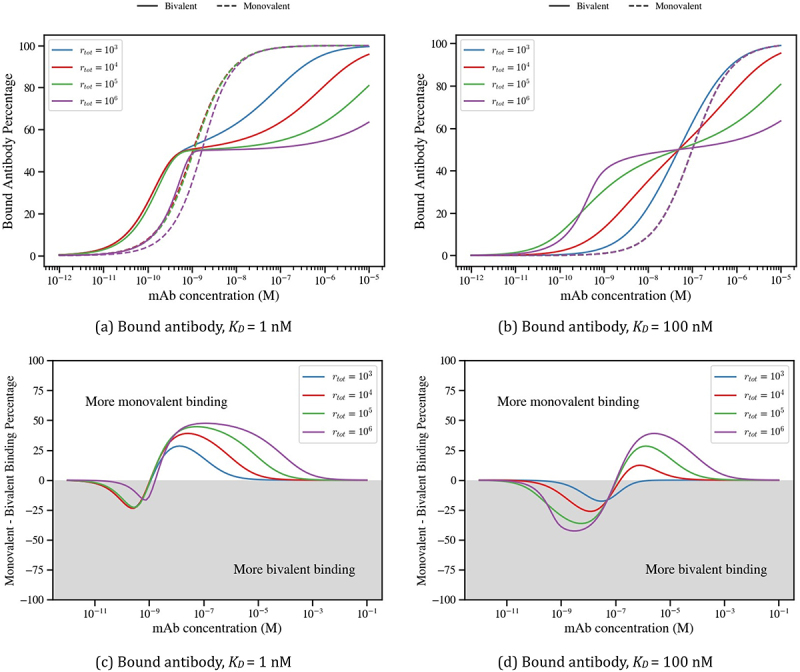
Simulations comparing bivalent and monovalent bound antibody percentages as antibody concentrations and antigen densities vary. Bivalent and monovalent model outputs, calculated as (A_1_+ A_2_)/r_tot_and A_1_/r_tot_, are shown as dashed and solid lines, respectively, in panels (a) and (b). Differences in bound antibody percentages between monovalent and bivalent antibodies is shown in panels (c) and (d). The white and gray panels indicate that the monovalent antibody has achieved more binding than the bivalent antibody, and conversely. In panels (a) and (c) we choose a binding affinity of K_D_ = 1 nM, while in panels (b) and (d) we fix K_D_ = 100 nM. All simulations assume an avidity binding rate of k_2_ = 10^−4^s^−1^.

To clarify the impact of avidity on the number of cell surface-bound antibody molecules, Figure 4 presents simulations of Equations (8) and (9) of bound antibody percentage, comparing monovalent ($A_{1}/r_{\mathrm{tot}}$) and bivalent ($\left(A_{1}+A_{2}\right)/r_{\mathrm{tot}}$) antibody binding across a range of antibody concentrations, $A_{\mathrm{init}}$, and antigen densities, $r_{\mathrm{tot}}$. Results are presented for a strong binder (*K*_*D*_ = 1 nM, Figure 4a,c) and a weak binder (*K*_*D*_ = 100 nM, Figure 4b,d). To further highlight the shift between monovalent and bivalent engagement as antibody concentration increases, we also plot the difference between the two binding modes, with gray and white regions representing more bivalent and monovalent binding, respectively (Figure 4c,d).

At low antibody concentrations ($A_{\mathrm{init}}\approx 10^{-12}-10^{-10}$ M), bivalent binding is prominent due to avidity. As antigen sites become saturated with bivalently bound antibody, e.g., when the bound antibody fraction is 50%, the number of monovalently bound antibodies surpasses that of bivalent ones. Complete saturation with monovalently bound antibody is achieved when $A_{\mathrm{init}}\approx 10^{-7}$ M.

As antibody concentration increases further, the total amount of bound antibody depends strongly on $r_{\mathrm{tot}}$. For higher antigen density ($r_{\mathrm{tot}}=10^{6}$), the plateau region of the bivalent antibody binding curve extends across a wider concentration range. Nevertheless, even at low antigen density ($r_{\mathrm{tot}}=10^{3}$), reaching comparable bound antibody levels requires a substantially higher concentration of bivalent antibodies relative to monovalent antibodies. This “avidity barrier” reduces the number of bound antibodies and is more prominent for large antigen densities.

The avidity barrier is also evident in Figure 4b for the case of a weaker affinity antibody (*K*_*D*_ = 100 nM). The key difference is the pronounced dependence on $r_{\mathrm{tot}}$ before antigens become saturated with antibody, with the proportion of bound antibody at intermediate concentrations (10^−10^–10^−8^ M) increasing with antigen density. This occurs because antigen binding is not in the tight-binding regime; the antibody-antigen affinity is not sufficiently strong relative to the antibody and antigen molecular abundances for binding to become stoichiometrically limited. As a result, antigen occupancy remains sensitive to antigen density and increases as $r_{\mathrm{tot}}$ increases. In contrast, when antigen binding occurs in the tight-binding regime (Figures 4a), binding becomes primarily limited by antibody availability rather than antigen density, causing binding curves for different antigen densities to collapse until antigen saturation is approached. This distinction is determined by the relative magnitudes of the binding affinity and the concentration obtained from the antigen density.^18^ It should also be noted that in the bivalent case, the *K*_*D*_ that dictates the tight-binding regime will be the apparent affinity arising from bivalent engagement and so will also be dependent on antigen diffusion.

Figure 4c,d further illustrate the interplay between monovalent and bivalent binding. In the low-concentration regime (10^−12^ −10^−8^M), bivalent binding dominates, particularly for weaker affinity antibodies. At higher concentrations (10^−8^ −10^−4^M), monovalent binding dominates, especially for higher antigen density. Finally, by simulating at antibody concentrations much larger than those typically seen within *in vitro* experiments (10^−4^ −10^−1^M), essentially all bivalent antibodies are bound monovalently. This confirms the presence of an avidity barrier: achieving equivalent levels of cell-bound antibody requires substantially higher antibody concentrations for the bivalent antibody compared to the monovalent format, with the magnitude of this barrier increasing as the antigen density increases.

### Avidity enhances antigen occupancy

**Figure 5.**
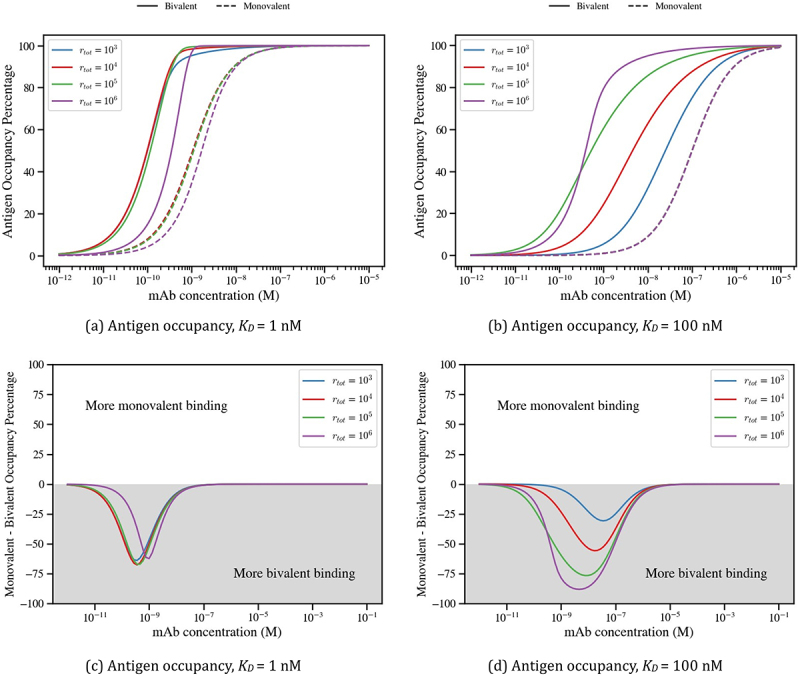
Simulations comparing bivalent and monovalent antibody antigen occupancy as antibody concentrations and antigen densities are varied. Bivalent and monovalent model outputs, calculated as (A_1_ + 2A_2_)/r_tot_ and A_1_/r_tot_, are shown as dashed and solid lines, respectively, in panels (a) and (b). Differences in antigen occupancy percentage between monovalent and bivalent antibodies are presented in panels (c) and (d). The white and gray panels indicate that the monovalent antibody has achieved more binding than the bivalent antibody, and conversely. Panels (a) and (c), respectively, show the percentage of bound antibody for a binding affinity of K_D_ = 1 nM, while panels (b) and (d) display the same quantities for K_D_ = 100 nM. All simulations assume an avidity binding rate of k_2_=10^−4^s^−1^.

To determine how avidity, antigen density, and binding affinity impact antigen occupancy, we compared the antigen occupancy of monovalent ($A_{1}/r_{\mathrm{tot}}$) and bivalent ($\left(A_{1}+2A_{2}\right)/r_{\mathrm{tot}}$) antibodies (Figure 5). Bivalent antibodies consistently achieved higher antigen occupancy than their monovalent counterparts, and this advantage persisted across the entire concentration range (Figure 5a,b). This contrasts the patterns observed in Figure 4a,b where monovalent antibodies achieved higher levels of cell-bound antibody at lower concentrations than their bivalent counterparts.

The influence of avidity is evident at all antigen densities and for both affinities, as the bivalent curves shift toward lower concentration ranges (10^−12^ −10^−10^M). Furthermore, the tight-binding limit is again apparent: under strong affinity (Figure 5a), the curves converge as available antigen is depleted, while under weaker affinity (Figure 5b), they remain distinct across different antigen densities. Figure 5a,b additionally show that affinity affects the dose-occupancy relationship, with the occupancy for the stronger affinity bivalent antibody plateauing at approximately 10^−10^ M for all antigen densities compared to the continued dose-dependence across the concentration range for the weaker affinity antibody. While initially this may seem to contradict the dose independence observed for in other studies,^19^ we believe this discrepancy may arise from nivolumab having a strong affinity for PD-1 along with the clinical concentrations used in their study potentially corresponding to the plateau region observed in Figure 5a.

Figure 5c,d underscore the advantage of avidity, with bivalent binding favored before antibody concentration is sufficiently large (10^−6^ M), to make bivalent antibodies bind monovalently. In contrast to the bound antibody simulations, these results predict that avidity is beneficial for antigen occupancy, rather than acting as a barrier.

### Impact of avidity on pharmacological quantities

To relate the results of the previous sections to pharmacological quantities of interest, we calculate the change in binding potency (defined as the concentration at which 50% of the maximum binding signal is obtained) for either bound antibody or antigen occupancy by setting *ϕ* =0.5 in Equations (21) and (22) between a monovalent and bivalent antibody and obtain

(25) $$\begin{array}{c} \mathrm{\Delta E}C_{50}^{bo\mathrm{und}}=\log_{10}\left(\frac{EC_{50}}{EC_{50}^{b\mathrm{ound}}}\right) \end{array}$$

(26) $$\begin{array}{c} \mathrm{\Delta E}C_{50}^{occ}=\log_{10}\left(\frac{EC_{50}}{EC_{50}^{occ}}\right) \end{array}$$

We also calculated $\Delta EC_{90}$ by setting *ϕ* =0.9 in Equations (21) and (22) to capture the change in near maximal response.

**Figure 6.**
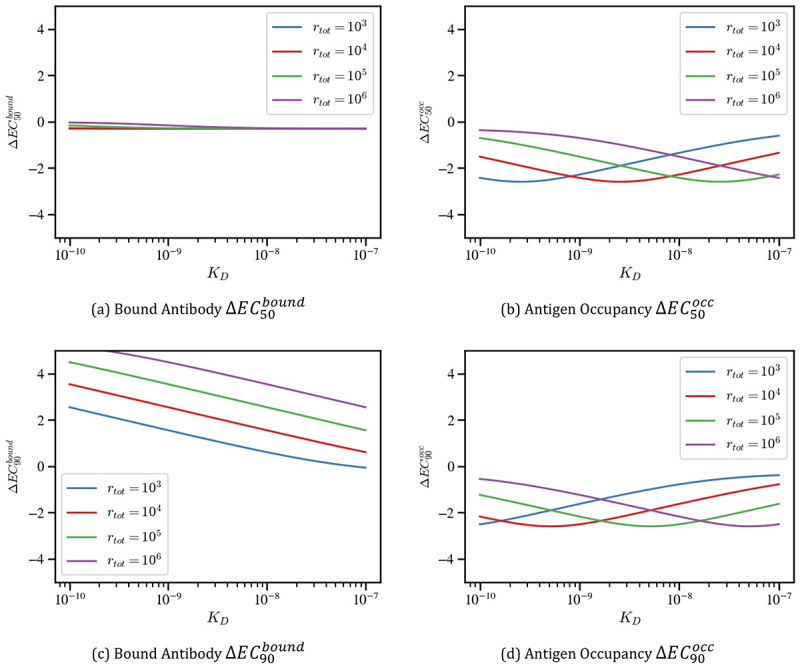
Plots of (a)$\mathrm{\Delta E}C_{50}^{bo\mathrm{und}}$, (b)$\mathrm{\Delta E}C_{50}^{occ}$, (c)$\mathrm{\Delta E}C_{90}^{bo\mathrm{und}}$ and (d)$\mathrm{\Delta E}C_{90}^{occ}$ for varying antigen densities and binding affinities as measured with K_D_.

Figure 6 shows that avidity affects pharmacological measures differently for bound antibody number and antigen occupancy. This distinction is especially pronounced for the $\Delta EC_{50}^{bo\mathrm{und}}$ and $\Delta EC_{50}^{occ}$ values (Figure 6a,b). Specifically, $\Delta EC_{50}^{bo\mathrm{und}}$ exhibits little dependence on both antibody affinity (*K*_*D*_) and antigen density ($r_{\mathrm{tot}}$), suggesting that avidity has minimal impact on the potency of drugs such as ADCs. In contrast, $\Delta EC_{50}^{occ}$ shows strong sensitivity to both parameters.

Figure 6b illustrates that the antigen density at which avidity has a pronounced impact (i.e., a large negative $\Delta EC_{50}^{occ}$) depends on antibody affinity. For strong binders (low *K*_*D*_), low antigen density ($r_{\mathrm{tot}}=10^{3}$) results in a substantial avidity effect. However, as *K*_*D*_ increases (weaker binding), higher antigen density leads to stronger avidity effects on *EC*_50_. Consequently, the optimum binding affinity for antagonists may depend on the antigen density.

While the curves for both $\Delta EC_{50}^{occ}$ and $\Delta EC_{90}^{occ}$ are qualitatively similar, the avidity barrier is most evident in the bound antibody measure $\Delta EC_{90}^{bo\mathrm{und}}$ (Figure 6c). Consistent with previous sections, Figure 6c shows that a monovalent antibody achieves 90% of maximum antibody opsonization at lower concentrations than a bivalent antibody, particularly at higher antigen densities. Notably, the influence of affinity is apparent: the magnitude of the avidity barrier decreases approximately linearly with increasing *K*_*D*_. This suggests that avidity imposes a large barrier to antibody opsonization when the antibody is a strong binder. Although avidity enhances antigen occupancy, it simultaneously hinders the accumulation of bound antibody in the high-affinity regime, and so may hinder the therapeutic effect of drugs such as ADCs.

To assess the robustness of our results to changes in the second binding rate, $k_{2}$, in supplementary Figure S1 we recreate Figure 6 for a select choice of $r_{\mathrm{tot}}$ while varying $k_{2}$ over a physically plausible range.^11^ Figure S1 shows some quantitative shift in $\Delta EC_{50}^{occ}$, $\Delta EC_{90}^{occ}$ and $\Delta EC_{90}^{bo\mathrm{und}}$ for certain $r_{\mathrm{tot}}$ values as $k_{2}$ varies, but no changes in the qualitative behavior of these quantities. Therefore, our conclusions are not dependent on the value of $k_{2}$.

### Calibrating the mathematical model with in vitro cell binding data highlights an affinity dependent bias

Next, we calibrated our mathematical model to *in vitro* on-cell binding data generated with antibodies and cell lines of varying affinities and antigen densities. To validate our mathematical model, *in vitro* on-cell binding data were generated for antibodies and cell lines of varying affinities and antigen densities. Antibody binding kinetics to human PD-1 were determined by SPR, and are presented in Table S3 of the supplementary information. Association and dissociation rates, in addition to derived K_*D*_ values, were similar among antibodies sharing anti-PD-1 CDRs, regardless of valency.

The density of cell surface human PD-1 was quantified using Quantum SimplyCellular beads, and was reported as antibody binding capacities (Table S2). These values correspond to the median antigen binding capacity in a normally distributed population. Such values are not the same as antigen density, since the antibody binding stoichiometry to both the beads and the cells is unknown. For the purposes of fitting the model, we only require an understanding of relative expression levels and, as such, we use the values of Table S2 as a proxy for $r_{\mathrm{tot}}$. As a consequence, results that involve $r_{\mathrm{tot}}$ should be interpreted as qualitative trends and relative changes across conditions, rather than quantitative changes in receptor density values.

We fitted the model to *in vitro* binding data obtained with the antibodies and cell lines listed in Tables S2 and S3. Parameters were estimated using Bayesian inference with *K*_*D*_ values from Table S3. The resulting marginal posterior parameter distributions are given in Supplementary Figures S2-S5.

**Figure 7.**
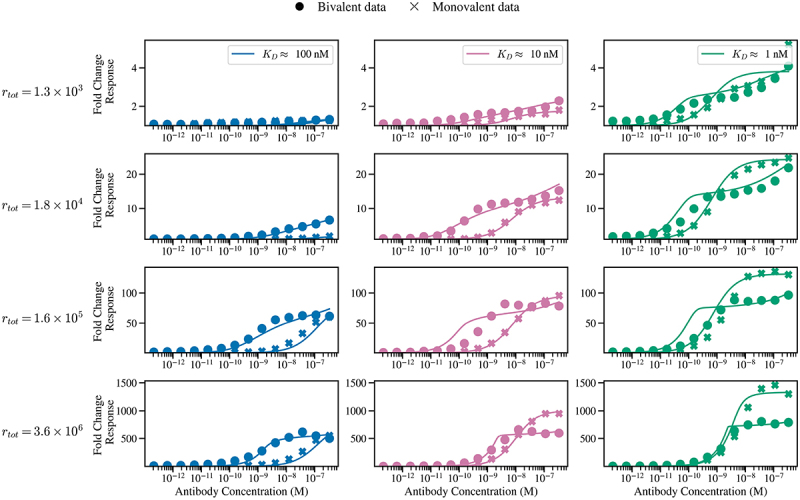
The solid lines are model fits to cell-binding data for the number of bound antibodies (A_1_ + A_2_) for each antibody clone and cell line (tables S2 and S3) using the means of the posterior parameter distributions given in Supplementary Figures S2-S5; circles and crosses denote bivalent and monovalent measurements, colored by clone. Signals were normalized to a no-antibody control. Rows indicate antigen expression levels (top to bottom: low to high), while columns and colors represent antibody clones arranged by affinity (left to right: weak to strong).

Figure 7 shows the results of fitting the model according to the relationship between experiments and model simulations outlined in Equation (24). Overall, the model closely reproduces the experimental data. A key feature is that the experimental data exhibits the avidity barrier for the strongest affinity clone (green curve): the bivalent antibody achieves markedly lower binding at high concentrations than its monovalent counterpart, and this effect diminishes as antigen density decreases.

The experimental data for the weaker affinity clones shows lower binding across all cell lines, even at high antibody concentrations, with this effect especially pronounced for the monovalent format. This is distinct from previous model simulations, where increasing antibody concentration was sufficient to overcome affinity differences and achieve binding levels comparable to high affinity clones (see Figure 4). We hypothesize that this discrepancy arises from the experimental wash step, in which antibody in solution is removed prior to addition of the secondary antibody and further incubation. This may reset the equilibrium, allowing cell-bound antibody to dissociate in solution and therefore not be observed in the experimental readout. This process would disproportionately affect poorly engaged antibodies, such as those with reduced valency or with weaker affinity, while bivalent antibodies would resist dissociation through bivalent avid engagement, matching the trend observed in the data.

**Figure 8.**
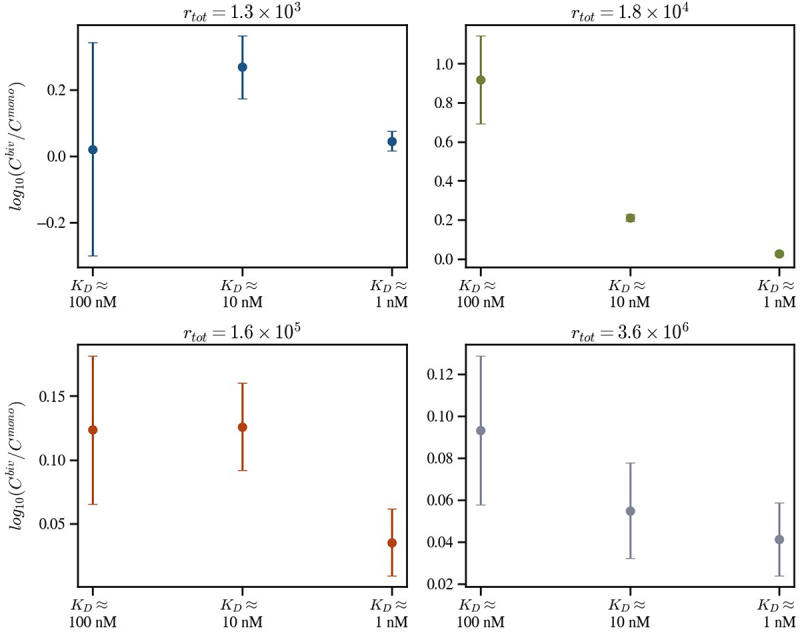
Comparison of the fitted posterior parameter distributions of **C**^biv^ and **C**^mono^, the constants for bivalent and monovalent antibodies that relate the model output to in vitro binding data for each PD-1 density (rtot) and K_D_. The dots represent the mean of the combined posterior distribution for log_10_(**C**^biv^/**C**^mono^). The error bars were also generated from the standard deviations of the combined distribution. Each plot corresponds to an individual cell line.

To test whether the model captures this effect, we compare the constants of proportionality, $C_{j}^{biv}$ and $C_{j}^{\mathrm{mono}}$. If monovalent antibodies are affected in the same way as bivalent antibodies during the assay, then $C_{j}^{biv}=C_{j}^{\mathrm{mono}}$ for all antibody clones. Figure 8 shows the ratio $\mathrm{lo}g_{10}\left(C_{j}^{biv}/C_{j}^{\mathrm{mono}}\right)$ across antibody clones and cell lines. Here, a positive value indicates that $C_{j}^{biv}>C_{j}^{\mathrm{mono}}$, suggesting loss of the monovalent experimental signal compared to the bivalent signal. Consistently, $C_{j}^{biv}>C_{j}^{\mathrm{mono}}$, except for the weakest affinity, lowest expression condition, where low signal-to-noise is likely to be dominant. Moreover, $\mathrm{lo}g_{10}\left(C_{j}^{biv}/C_{j}^{\mathrm{mono}}\right)$ negatively correlates with affinity, further supporting the interpretation that weaker affinity clones may preferentially dissociate during washing.

**Figure 9.**
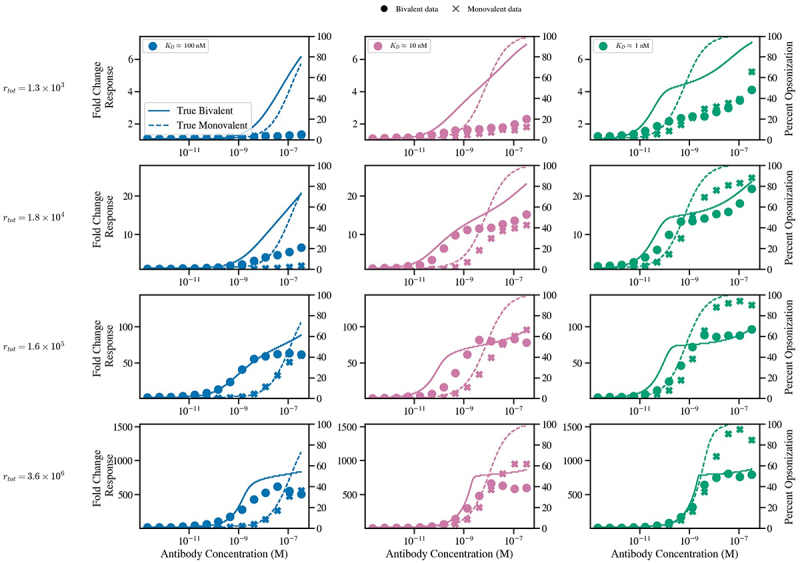
Comparison between experimental and model predicted “true” cell surface bound antibody data for each cell line and antibody clone where the ‘’true” simulations are obtained by setting **C**^biv^=**C**^mono^ = 1. The left y-axis measured the experimentally detected change in binding signal vs a background baseline while the right y-axis displays the model predicted antibody opsonization measured as (A_1_+ A_2_)/r_tot_ (A_1_/r_tot_in the monovalent case).

The results of Figure 8 indicate that *in vitro* antibody binding data does not always faithfully represent on-cell opsonization, with discrepancies depending on affinity, antibody format, and antigen density. To illustrate this, Figure 9 overlays the fitted model predictions of antibody opsonization, where $C^{\mathrm{biv}}=C^{\mathrm{mono}}=1$ for all antibodies and cell lines, with the experimental data. As expected, the weakest-affinity clones and the monovalent formats show the greatest divergence between measured binding and model predicted “true” opsonization.

Notably, the bivalent format also deviates substantially for the lowest-expressing cell line, which we attribute to predominantly monovalent engagement under conditions where sparse antigen density prevents simultaneous binding of both arms. In summary, the experimental data exhibits the model-predicted avidity barrier. Further, by calibrating the model to this data, we find evidence of systematic biases in experimentally measured binding, suggesting that true opsonization is strongly affinity-dependent and may often be underestimated in *in vitro* assays.

## Discussion

Antibody-based therapies are a diverse class of drugs that have proven successful across multiple diseases and indications. However, avidity associated with the bivalent nature of these molecules is not modeled with sufficient accuracy to enable translation of *K*_*D*_ values obtained from solution-based methods to *EC*_50_ values that might be observed in *in vitro* on-cell binding assays. In this study, we showed how the bivalency and avidity of an antibody impact its on-cell antigen binding and postulated the potential downstream therapeutic effects. We focused on the impact of avidity on antigen occupancy and bound antibody number, quantities directly linked to the therapeutic performance of antibody antagonists and immune cell engagers, respectively, and used an existing mathematical model of monospecific bivalent antibody–antigen binding.^11^ Model simulations revealed an “avidity barrier” for on-cell antibody opsonization, whereby monovalent antibodies achieve higher levels of cell-bound antibody than their bivalent counterparts at high concentrations (Figure 4). This effect is most pronounced under high antigen densities, where the likelihood of simultaneous engagement of both binding arms increases. The model suggests that bivalently bound antibodies achieve only half the maximum binding of their monovalent counterparts. Strong bivalent engagement effectively out-competes free antibody for binding, resulting in a 2:1 antigen:antibody ratio. As this avidity is overcome, and monovalent binding begins to compete, the ratio shifts closer to 1:1. With a greater density of antibodies per cell, it would follow that there is a greater density of immunomodulatory Fc domains per cell, which may enhance effector function, analogously to how cells possessing greater antigen density are more susceptible to antibody effector functions. One might also exploit this to design novel therapeutic antibody formats to achieve increased cytotoxic payload delivery per antigen.

In contrast, simulations of antigen occupancy exhibited consistently greater binding for bivalent antibodies across all concentrations and expression levels (Figure 5). Avidity thus exerts a dual effect: enhancing antigen occupancy while simultaneously limiting the number of antibodies bound per cell. This dual effect is further reflected in predicted pharmacological metrics: for opsonization, monovalent antibodies display markedly improved *EC*_90_ values relative to bivalent antibodies, whereas for antigen occupancy, both *EC*_50_ and *EC*_90_ favored the bivalent format (Figure 6).

We next calibrated our model predictions using a panel of monovalent and bivalent anti-PD-1 antibodies across cell lines with varying PD-1 density. The *in vitro* opsonization binding data qualitatively reproduced the predicted avidity barrier, especially for the highest-affinity clones (Figure 7). Parametrizing the model with these data, we revealed an affinity and format dependent bias, whereby weaker affinity and monovalent antibodies exhibited lower experimentally measured binding than stronger affinity and bivalent counterparts (Figure 9). We predict that this discrepancy is due to assay wash steps, with free antibody removal resetting the system equilibrium. Under these conditions, weaker affinity and monovalently bound antibodies may dissociate more rapidly from the cell surface than bivalent counterparts that are partially stabilized by avidity. This highlights an important distinction between model predictions, which assume system equilibrium, and empirical measurements, which are influenced by assay design. In practice, cell-binding protocols unavoidably involve multiple wash steps prior to detection with a secondary fluorescent antibody. Each wash could disproportionately reduce binding of weaker and monovalent antibodies, causing underestimates of the “true” extent of on-cell opsonization for some formats and affinities. There are no current physiologically relevant binding approaches that can mimic *in vivo* binding, nor analytical techniques that can measure *in situ* binding in the presence of bulk non-associated antibody. Therefore, a combination of both *in vitro* experiments and mathematical modeling may enhance the accuracy of antibody binding studies.

### Avidity and antibody presentation

The stoichiometry of antibody-cell surface antigen binding depends on the ratio of antibody to antigen. At low antibody-to-antigen ratios, bivalent engagement dominates. Under these conditions, binding of a second antigen by a cell-bound antibody is faster than the corresponding in-solution reaction, producing stable 2:1 complexes. As the antibody-to-antigen ratio increases, binding transitions from bivalent to monovalent as there is sufficient free antibody in-solution to outcompete binding of the second arm.

This change in stoichiometry increases the number of Fc and other immune-engaging domains per antigen. Maximizing Fc domains per cell might be expected to enhance Fc*γ*R cross-linking and associated immune effector functions such as antibody-dependent cellular cytotoxicity and antibody-dependent cellular phagocytosis, in addition to those not mediated by Fc*γ*Rs such as complement-dependent cytotoxicity. Additionally, in a landscape increasingly dominated by T cell engagers and ADCs, maximizing anti-CD3 domains and cytotoxic payloads per cell may confer therapeutic benefit. Additional data from such functional experiments would allow extension of the model to predict efficacy and potency from kinetic parameters. Furthermore, binding of an antigen by the second arm of an antibody involves diffusion and the clustering of antigens, potentially altering their location, orientation, and subsequent binding. While in previous work we have shown that diffusion is the rate-limiting step in this reaction,^11^ orientation may play a role in limiting bivalent engagement, particularly at large antigen densities. As such, a potential model extension is to explicitly model all components of the binding of the second arm.

### Avidity as a strategy to enhance apparent affinity

When binding is measured as antigen occupancy, rather than antibodies per cell, the benefit of avidity is apparent. The apparent affinity of an antibody for its antigen is greater than the sum of two single Fab affinities, indicating that the engagement of one arm acts to stabilize the complex and promotes the binding of the second arm through increased local concentration. If true, this would suggest that engineering the molecule to append additional Fab domains may confer higher-order avidity with resulting higher apparent affinity. For cell surface antigens where antibody discovery is challenging and sufficient affinity cannot be reached, using avidity to enhance binding may present a viable strategy to achieve necessary target engagement. However, clustering of cell surface receptors through avid engagement of antibodies may induce undesirable receptor agonism; thus, functional consequences of higher-order engagement should be monitored closely.

### Clinical relevance

The work presented here demonstrates that both CDR affinity for the target antigen, and avid binding by crosslinking antigens on the cell membrane, influence the concentration-effect relationship for a given monoclonal antibody. This is important because there are constraints on the concentration of antibody that can be achieved in a given tissue *in vivo*. First, there are constraints on the dose size, dictated by formulation requirements (especially in the case of subcutaneous dosing) and, to some extent, cost. Second, there is the restricted distribution of mAbs into tissue due to slow extravasation. An active concentration must be maintained in that tissue during a period of time in each dosing interval. For a receptor antagonist, this might need to be maintained for the entire dosing interval. Otherwise, for antibody-mediated cell death, transient depletion may suffice assuming target cells do not repopulate too rapidly. Pharmacokinetics will determine whether the required duration of tissue exposure is feasible and the level of potency required.

Understanding how affinity and avidity influence the potency of a mAb then informs molecular design. In particular, the results of this work suggest that in some contexts opsonization might be maximized by avoiding the avidity barrier with a monovalent molecule.^3,4^ The converse is true of a receptor antagonist where avidity enhances occupancy at low mAb concentrations. A practical consideration is that the epitope may impact the extent of avidity, reducing the ability to cross-link by introducing spatial constraints.

The monovalent antibodies characterized here were engineered to isolate valency as a parameter while retaining an IgG-like structure, but otherwise do not represent a therapeutic format that is likely to be explored in the clinic. However, the benefits of valency-engineered antibody-based therapeutics have already been reported for single-chain variable fragment- and Fab-derived modalities, such as bispecifc T cell engagers, and novel fragment drug conjugates.^20,21^ Beyond engineering antigen-valency, one might also question whether shifting the balance through other means (e.g., by increasing effector domain, Fc*γ*R or anti-CD3 valency), might result in similar therapeutic benefit. In other words, mimicking the 1:1 Fc:antigen ratio present in monovalent antibodies by matching the number of effector domains to the number of binding domains may confer therapeutic benefit while retaining favorable antigen avidity.

The model shows how exploiting avidity can reduce the dose needed to achieve the target engagement required for a given therapeutic effect. This comes at the cost of reducing the total quantity of antibody and associated effector domains bound to the cell surface due to intermolecular competition for antigen binding. A novel way to retain avidity while reducing competition is to use biparatopic antibodies, which bind unique epitopes on the surface of an antigen. Such molecules have demonstrated superior binding capacity compared to the bivalent antibodies from which they were derived.^22^

### Future work and conclusion

This study shows how a mathematical model for bivalent antibody-antigen binding can be used to study the impact of avidity on target antigen engagement, in the context of cell surface PD-1. Avidity can enhance or hinder therapeutic potential depending on whether antigen occupancy or antibody opsonization governs efficacy and potency. The model predictions were calibrated against *in vitro* opsonization data that also highlighted affinity and valency-dependent bias in on-cell experimental binding, which we hypothesize is due to the assay wash step.

This model clarifies the relationship between SPR-derived estimates of *K*_*D*_ and flow cytometry derived estimates of *EC*_50_, and explains why it is important to differentiate between the two values. *EC*_50_ is a function of avid binding and assay limitations, including but not exclusively tight-binding and ligand depletion, while *K*_*D*_ is a function of the chemical binding between a single epitope and Fab binding arm.

Further work in diverse antigen and epitope contexts is needed to determine whether this model universally describes cell surface antigen engagement. A change in epitope or antigen might be expected to alter the propensity of an antibody to engage avidly, manifesting as an altered value of *k*_2_, which would serve as an “avidity measure” and vary depending on antigen diffusion and epitope display.

With a mathematical model for antibody engagement established, future work could integrate this binding with downstream effector function, by incorporating the formation of ternary complexes with Fc*γ*Rs, and internalization rates of antibody:antigen complexes for ADCs. Collectively, such a model could serve as a powerful tool to predict functional efficacy of antibody-based therapeutics from a small set of simple parameters, thus providing a quantitative link between molecular binding kinetics and therapeutic efficacy.

## Supplementary Material

Supplementary_Information_for_Avidity_barrier_manuscript_revised.pdf

## Abbreviations

PD-1: Programmed cell death protein 1
ADC: Antibody drug conjugates
Fc: Fragment crystallizable
Fc*γ*R: Fc gamma receptor
CD3: Cluster of differentiation 3
NKp30: Natural killer protein 30
CDR: Complementarity-determining region
SPR: Surface plasmon resonance
mAb: Monoclonal antibody
IgG: Immunoglobulin G
ODE: Ordinary differential equation
CHO: Chinese hamster ovary
Fab: Fragment antigen-binding
mRNA: Messenger ribonucleic acid
EF1*α*: Elongation factor 1-alpha 1
PGK: Phosphoglycerate kinase
SV40: Simian virus 40
DNA: Deoxyribonucleic acid
APC: Allophycocyanin
MFI: Median fluorescence intensity
VH: Variable heavy
VL: Variable light
HEK293: Human embryonic kidney 293
RPM: Revolutions per minute
PBS: Phosphate-buffered saline
HBS: HEPES buffered saline
EDTA: Ethylenediaminetetraacetic acid
MCMC: Markov chain Monte Carlo
