# Correction

**DOI:** 10.1080/19420862.2026.2686538

**Published:** 2026-06-09

**Article title**: The double-edged nature of antibody bivalency: Mathematical and experimental analysis of cell surface antigen occupancy and opsonization

**Authors**: Heirene, L., Lodge, J., Fedorova, M., Gauntlett, R., Cordy, J., Rattray, Z., Byrne, H., Gaffney, E., & Yates, J. W. T.

**Journal**: *mAbs*

**DOI**: https://doi.org/10.1080/19420862.2026.2681843

The author has identified an error in the caption of Figure 2, which was incorrectly published in the article.

The author has requested that the current caption of Figure 2 be replaced with the updated version provided below, as it more accurately reflects the original intent.

**Figure 2**: A schematic of antigen occupancy and Fc domain presentation following monovalent or bivalent antibody engagement. (a) Antibodies engaging antigens monovalently can achieve greater density and potentially achieve enhanced FcγR clustering. (b) In contrast, bivalently engaged antibodies can only present half the number of Fc domains to immune effector cells.

