# Supplementary material for: The double-edged nature of antibody bivalency: mathematical and experimental analysis of cell surface antigen occupancy and opsonization
Source: MAbs. 2026 Jun 3;18(1):2681843. doi: 10.1080/19420862.2026.2681843 (PMC13240941; doi:10.1080/19420862.2026.2681843)
Supplement: Supplementary_Information_for_Avidity_barrier_manuscript_revised.pdf [file KMAB_A_2681843_SM7531.pdf]

Supplementary Information for The Double-Edged Nature of Antibody Bivalency: Mathematical and Experimental Analysis of Cell Surface Antigen Occupancy and Opsonization

Luke Heirene[1,*], James Lodge[2,4,*], Marina Fedorova[2], Ross Gauntlett[2,4], Joanna Cordy[2], Zahra Rattray[4], Helen Byrne[1], Eamonn Gaffney[1], and James W.T. Yates[3]

[1]Mathematical Institute, University of Oxford

[2]Large Molecule Research, GSK

[3]DMPK, Preclinical Sciences, GSK

[4]Strathclyde Institute of Pharmacy and Biomedical Sciences, University of Strathclyde

[*]These authors contributed equally to this work.

S1 Model Assay Parameter Table

Table S1: Model assay parameters associated with main text Equations (8)-(9)

Parameter	Definition	Estimated Value (units)
T^0	Target cell number in assay	2×10^4 (cells)
V_{well}	Assay reaction well volume	25 (μL)
$\sigma = V_{\text{well}} Na/T^0$	Concentration to protein number conversion factor	7.5×10^{14} ($\text{M}^{-1}\text{cell}^{-1}$)
Na	Avogadro constant	6.02214×10^{23} (mol^{-1})

S2 Derivation of Pharmacological Quantities

Here, we derive the steady state values of the pharmacological quantities introduced in Section 2.1.1 within the main manuscript. In particular, we seek expressions for the concentration at which the fraction ϕ of maximal bivalent antibody binding is achieved when either measuring binding as antigen occupancy or bound antibody number (opsonisation), defined as EC_{ϕ}^{occ} and EC_{ϕ}^{bound} respectively.

Using EC_{ϕ}^{bound} as an example, as we are interested in the steady state values of these quantities, we begin by setting $\mathrm{d}/\mathrm{d}t = 0$ in Equations (8) and (9) of the main text. Furthermore, we make the substitution $\phi r_{\text{tot}} = A_1 + A_2$ as we look to find the concentration at which bound antibody number is ϕ percent of

the maximum r_{tot}:

$$\begin{aligned}0 &= 2k_1(A_{\text{tot}} - \phi r_{\text{tot}})(r_{\text{tot}}[1-\phi] - A_2) - k_{\text{off}}(\phi r_{\text{tot}} - A_2) \\ &\quad - k_2(\phi r_{\text{tot}} - A_2)(r_{\text{tot}}[1-\phi] - A_2) + 2k_{\text{off}}A_2,\end{aligned} \tag{1}$$

$$0 = k_2(\phi r_{\text{tot}} - A_2)(r_{\text{tot}}[1-\phi] - A_2) - 2k_{\text{off}}A_2. \tag{2}$$

Next, SI Equation (2) can be rearranged into a quadratic in A_2 for which the only root that gives a feasible value of $0 \leq A_2 \leq r_{\text{tot}}/2$ is given by

$$A_2 = \frac{r_{\text{tot}} + \frac{2k_{\text{off}}}{k_2} - \sqrt{(r_{\text{tot}} + \frac{2k_{\text{off}}}{k_2})^2 - 4\phi r_{\text{tot}}^2(1-\phi)}}{2} =: f_1(\phi). \tag{3}$$

Adding SI Equations (1) and (2), substituting $f_1(\phi)$ for A_2 in SI Equation (1) and rearranging for the antibody concentration, $A_{\text{init}} = A_{\text{tot}}/\sigma$, we arrive at

$$A_{\text{init}} = \frac{K_D}{2}\left(\frac{\phi r_{\text{tot}} - f_1(\phi)}{r_{\text{tot}}(1-\phi) - f_1(\phi)}\right) + \frac{\phi r_{\text{tot}}}{\sigma} =: EC_{\phi}^{bound}, \tag{4}$$

where $K_D = k_{\text{off}}/k_{\text{on}} = k_{\text{off}}/(k_1\sigma)$. An analogous expression when measuring binding with antigen occupancy, defined as EC_{ϕ}^{occ}, can be obtained by following the same procedure as above but instead making the substitution $\phi r_{\text{tot}} = A_1 + 2A_2$:

$$EC_{\phi}^{occ} := \frac{K_D}{2}\left(\frac{\phi r_{\text{tot}} - 2f_2(\phi)}{r_{\text{tot}}(1-\phi)}\right) + \frac{\phi r_{\text{tot}} - f_2(\phi)}{\sigma}, \tag{5}$$

where $f_2(\phi)$ is defined as

$$f_2(\phi) = \frac{\phi r_{\text{tot}}^2(1-\phi)}{\frac{2k_{\text{off}}}{k_2} + 2r_{\text{tot}}(1-\phi)}. \tag{6}$$

In the case of monovalent antibody-antigen binding as described by main text Equation (11), setting $\mathrm{d}/\mathrm{d}t = 0$ and substituting $\phi r_{\text{tot}} = A_1$ we obtain

$$EC_{\phi} = \phi\left(\frac{K_D}{1-\phi} + \frac{r_{\text{tot}}}{\sigma}\right). \tag{7}$$

In the main text, we set ϕ to 0.5 or 0.9 depending on the quantity of interest.

S3 Dependence of Pharmacological Quantities on k_2

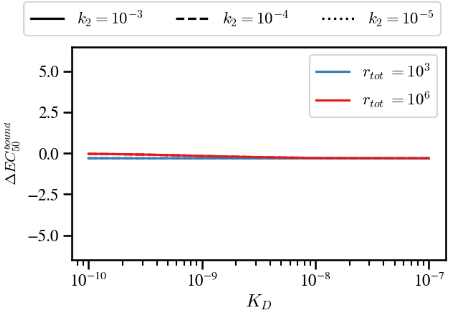

(a) Bound Antibody ΔEC_{50}^{bound}

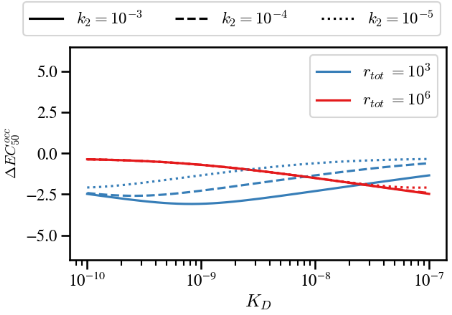

(b) Antigen Occupancy ΔEC_{50}^{occ}

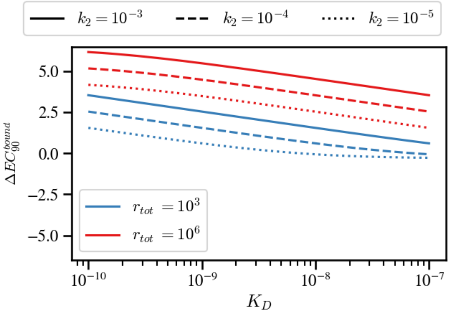

(c) Bound Antibody ΔEC_{90}^{bound}

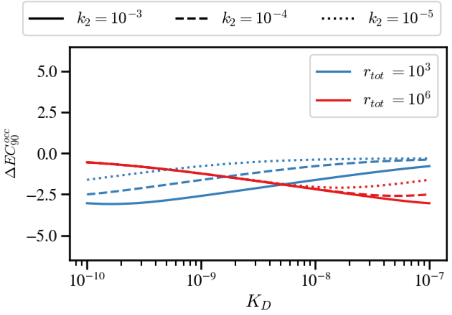

(d) Antigen Occupancy ΔEC_{90}^{occ}

Figure S1: Plots of (a) ΔEC_{50}^{bound}, (b) ΔEC_{50}^{occ}, (c) ΔEC_{90}^{bound} and (d) ΔEC_{90}^{occ} for varying antigen densities, k_2 values and binding affinities as measured with K_D. The range of k_2 values is obtained from Heirene et al. 2025.

S4 Posterior Parameter Distributions

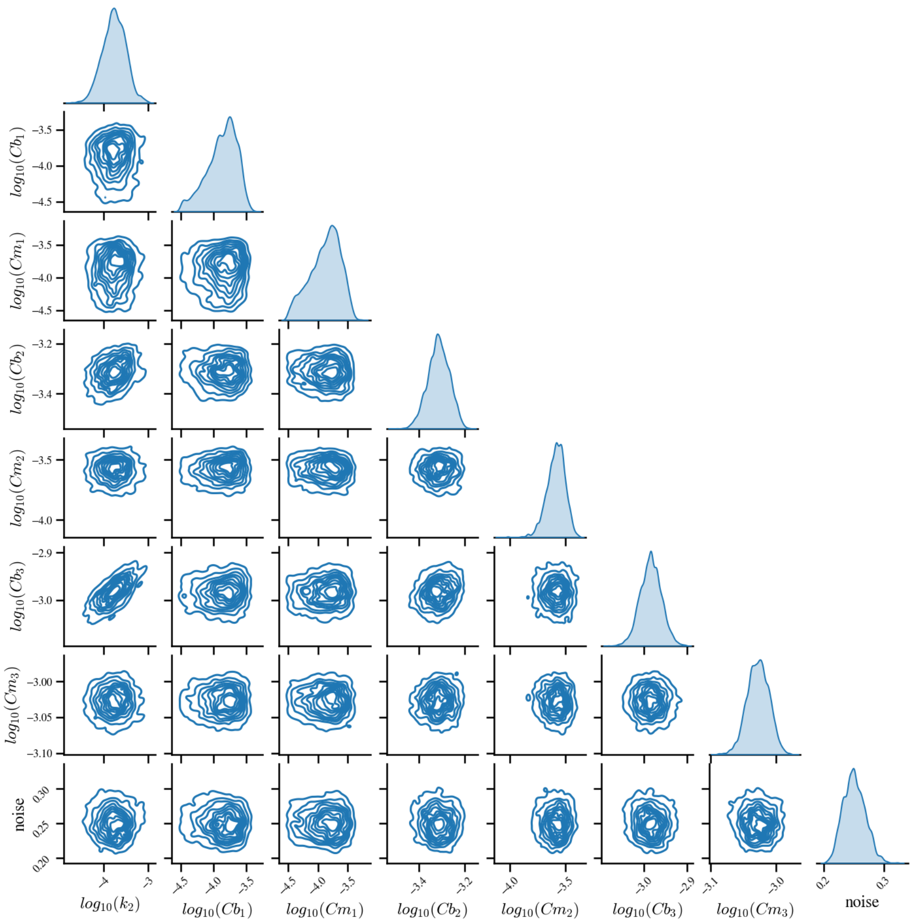

Figure S2: Marginal posterior distributions generated as described in main text Section 2.7 using the PINTS package during model inference for the cell line with low antigen expression.

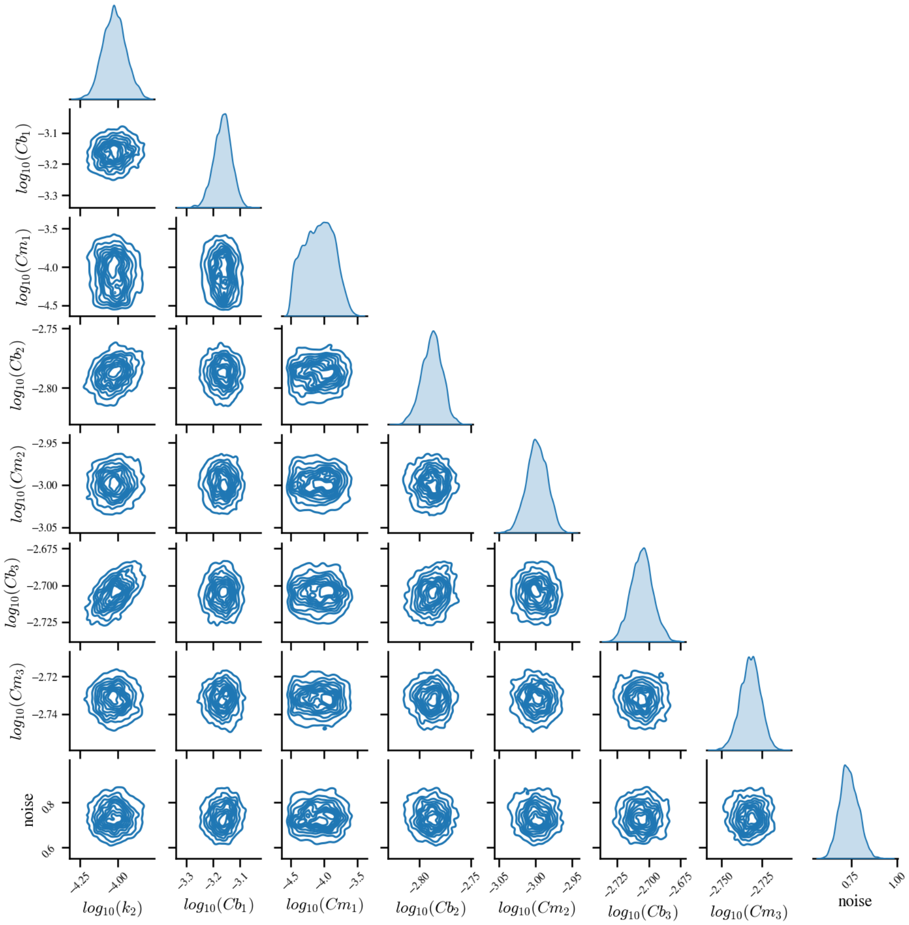

Figure S3: Marginal posterior distributions generated as described in main text Section 2.7 using the PINTS package during model inference for the cell line with medium antigen expression

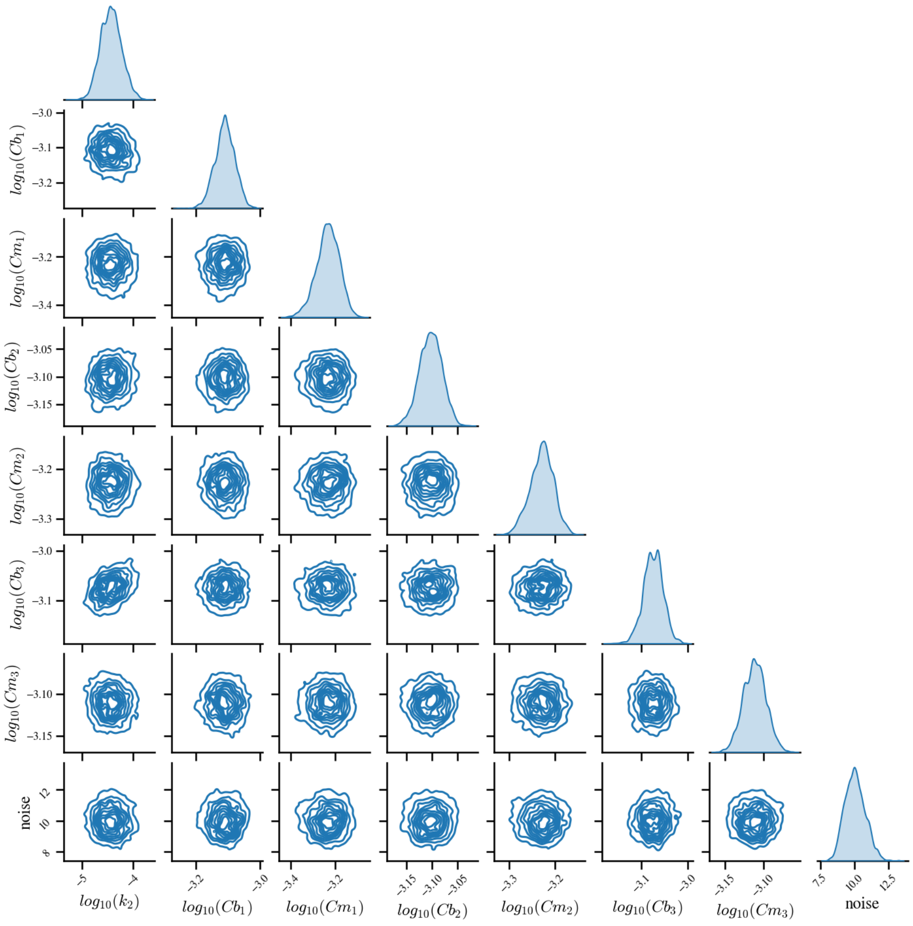

Figure S4: Marginal posterior distributions generated as described in main text Section 2.7 using the PINTS package during model inference for the cell line with high antigen expression

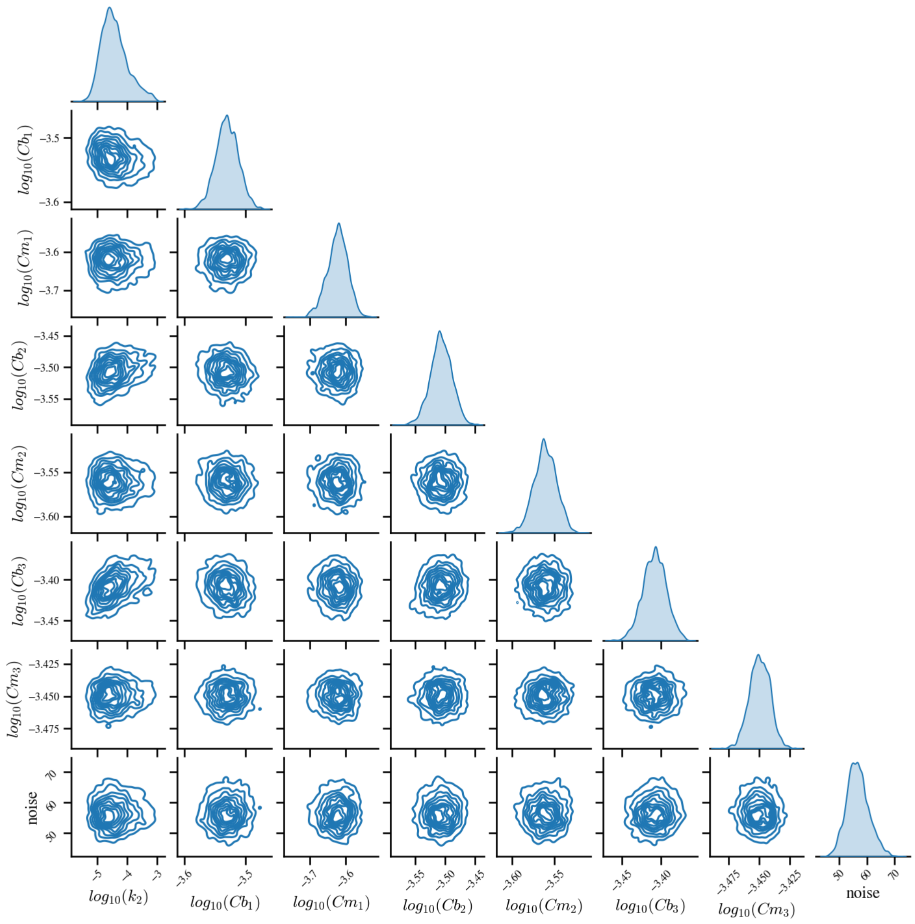

Figure S5: Marginal posterior distributions generated as described in main text Section 2.7 using the PINTS package during model inference for the cell line with very high antigen expression

S5 Experimentally-derived values for modelling

Table S2: Antibody binding capacities of recombinant CHO cell lines, as determined using SimplyCellular quantification beads, to two significant figures. Reported values are the mean and standard deviation of three replicate experiments.

**Cell Line Name**	**Antibody binding capacity (SD)**
**V. High**	3.6×10^6 (4.2×10^5)
**High**	1.6×10^5 (1.8×10^4)
**Medium**	1.8×10^4 (7.9×10^3)
**Low**	1.3×10^3 (4.3×10^2)

Table S3: Kinetic rate constants and affinity values determined by surface plasmon resonance as detailed in Section 2.5. Reported values are the mean of three replicate sets of sensorgrams, with standard deviation reported in brackets.

**Antibody ID**	**PD-1 Valency**	$k_{\mathbf{on}}$ ($\mathbf{M^{-1}s^{-1}}$)	$k_{\mathbf{off}}$ ($\mathbf{s^{-1}}$)	K_D (**M**)
**Strong**	**Bivalent**	$1.75{\times}10^{6}$ ($2.62{\times}10^{5}$)	$1.01{\times}10^{-3}$ ($4.98{\times}10^{-5}$)	$5.85{\times}10^{-10}$ ($6.13{\times}10^{-11}$)
	**Monovalent**	$1.73{\times}10^{6}$ ($1.28{\times}10^{5}$)	$1.09{\times}10^{-3}$ ($1.12{\times}10^{-4}$)	$6.32{\times}10^{-10}$ ($6.18{\times}10^{-11}$)
**Medium**	**Bivalent**	$1.48{\times}10^{6}$ ($4.91{\times}10^{4}$)	$1.37{\times}10^{-2}$ ($1.23{\times}10^{-3}$)	$9.26{\times}10^{-9}$ ($5.43{\times}10^{-10}$)
	**Monovalent**	$1.22{\times}10^{6}$ ($4.28{\times}10^{4}$)	$9.35{\times}10^{-3}$ ($7.40{\times}10^{-4}$)	$7.67{\times}10^{-9}$ ($4.72{\times}10^{-10}$)
**Weak**	**Bivalent**	$8.85{\times}10^{5}$ ($3.20{\times}10^{5}$)	$9.76{\times}10^{-2}$ ($3.36{\times}10^{-2}$)	$1.11{\times}10^{-7}$ ($1.24{\times}10^{-8}$)
	**Monovalent**	$8.42{\times}10^{5}$ ($2.65{\times}10^{5}$)	$1.08{\times}10^{-1}$ ($2.02{\times}10^{-2}$)	$1.31{\times}10^{-7}$ ($1.53{\times}10^{-8}$)
